# Seroprevalence of HHV-6 and HHV-8 among blood donors in Greece

**DOI:** 10.1186/1743-422X-11-153

**Published:** 2014-08-27

**Authors:** Marianna Politou, Dimitrios Koutras, Georgios Kaparos, Serena Valsami, Theodoros Pittaras, Emmanouil Logothetis, George Panayiotakopoulos, Evangelia Kouskouni

**Affiliations:** Blood Transfusion Department, Aretaieion Hospital, Athens University Medical School, Athens, Greece; Microbiology Laboratory ARETAIEION Hospital, Athens University Medical School, Athens, Greece; Pharmacology Department, Medical School, University of Patras, Patras, Greece

**Keywords:** HHV-6 seroprevalence, HHV-8 seroprevalence, Blood donors, Greece

## Abstract

**Background:**

Herpes viruses infection transmitted through healthy but infected blood donors pose a danger to herpes-naive immunocompromised recipients. The risk of transfusion-related HHV-8 transmission is different in endemic and not endemic areas. HHV-6 and HHV-8 seroprevalence and viral load among blood donors have been reported from different countries. The aim of our study was to assess the seroprevalence of HHV-8 and HHV-6 in volunteer blood donors from Greece which is unknown.

**Findings:**

Serum samples from 179 healthy blood donors were tested for the presence of IgG antibodies against HHV-6 and HHV-8 with ELISA. None of the 179 donors of Greek origin tested was positive for HHV-8. HHV-6 seropositivity was assessed in 160 blood donors’ samples and was found to be 78.75% (126/160). The HHV-6 seroprevalence did not differ either between males and females or among different decade age groups.

**Conclusions:**

The fact, that no blood donor was positive for HHV-8 IgG antibodies indicates that the risk for transfusion related HHV-8 transmission in Greece, if any, is negligible and does not warrant broad testing for HHV-8. Definitely further studies are needed, in order to clarify the potential risk of HHV-6 transmission.

## Findings

### Introduction

Herpes viruses 6 and 8 (HHV-6 and HHV-8) are enveloped ds-DNA viruses that can remain latent in the host cells for a long time. They can re-enter a lytic cycle and thus cause reactivation of the viral infection. Herpes viruses i transmitted from healthy but infected blood donors pose the danger of primary infection to herpes-naive immunocompromised recipients. HHV-8 seroprevalence has been assessed in several countries and it is found that the risk of transfusion-related HHV-8 transmission is different in endemic and not endemic areas [1]. I HHV-6 seroprevalence among blood donors has also been reported from different countries [2]. The aim of the present study was to assess the prevalence of anti- HHV-8 and anti-HHV-6 antibodies in volunteer blood donors in Greece, which is unknown.

## Materials and methods

We analyzed 179 blood donor samples collected from January 2012 to May 2012 in the Blood Bank of Aretaieion University Hospital, in Athens. All donors completed the blood donor screening questionnaire and were eligible for blood donation. Serum samples were stored at -20°C prior to being tested. The study was approved by the Scientific and Ethics Committee of our Hospital, and informed consent was obtained from all participants.

All donors underwent the routine Blood Bank screening that includes HBsAg testing, antibodies against HIV 1/2, HCV and HTLV1/2, *VDRL* (Venereal Disease Research Laboratory) test, along with Nucleic Acid Testing (NAT) for HBV, HCV, and HIV. We analyzed the data only from blood donors, who tested negative for the full above screening.

The anti-HHV-8 and anti-HHV-6 lytic IgG antibodies were analysed in serum samples using commercially available ELISA kits (Advanced Biotechnologies Inc HHV-8 IgG., Columbia, MD, USA and PANBIO HHV-6 IgG Inverness Medical Innovations Australia), according to the manufacturer’s instructions.

All statistical analysis was performed using SPSS v.22.0 (IBM Corp, Armonk, NY, USA) P values were considered as significant when P < 0.05.

## Results

The study group comprised of 179 volunteers blood donors, 136 males and 43 females with a mean age of 38 years (range 19–64 years). All tested negative for HBV, HCV, HIV1/2, HTLV1/2 and VDRL.

None of the 179 donors of Greek origin tested was positive for HHV-8. There was only one additional sample which tested positive for anti-HHV-8 antibodies from one Greek male donor, who tested positive for HIV (both with Elisa and NAT) and thus was excluded from the study.

The seroprevalence of HHV6 was assessed in 160 blood donor samples and was found to be 78.75% (126/160). There was no significant difference of seropoprevalence neither between males (77.68%: 94/121) and females (82.05%: 32/39) nor among different decade age groups (82.61% group A: 19–30 years old, 81.82% group B: 31–40 years old, 69.76% group C: 41–50 years old, 81.48% group D: 51–64 years old) (Table 1).

**Table 1:** HHV-6 seroprevalence (sex and age distribution)*

	Age groups (years)				Positive cases		Total
	A: 19-30 N = 46	B: 31-40 N = 44	C: 41-50 N = 43	D: 51-64 N = 27			
Males	37	34	32	18	94	p = 0.657	121
Females	9	10	11	9	32		39
Positive cases	38 (82.61%)	36 (81.82%)	30 (69.76%)	22 (81.48%)	126 (78.75%)		160
	p = 0.526	p = 0.668	p = 0.126	P = 0.802			

*p for each age group was calculated versus the other age groups.

## Discussion

Epidemiological data for HHV-6 and HHV-8 in blood donor in Greece is poor. HHV-8 is the etiological agent for Kaposi sarcoma, Castelmann’ disease and primaty effusion lymphoma. Kaposi Sarcoma unrelated to HIV infection is more common in countries of Mediterranean “basin” such as Greece and Italy than in Northern Europe and the USA [3].

The seroprevalence of HHV-8 in blood donors in Southern Europe ranges from 4.5% (9/200) in Spain Basque country to 20% and 25% in Sicily and Sardinia respectively. Interestingly, a seroprevalence of 2.4% was reported recently in Eastern Sicily [4]. However, the seroprevelance in blood donors in the U.K. (2.7%) and the United States (1.4%) is significantly lower [5, 6].

Since HHV-8 modes of transmission are elusive and the HHV-8 transmission through blood transfusion differs in endemic and non endemic areas, assessing HHV-8 seroprevalence among blood donors in Greece would help us identify the putative danger of transfusion related HHV-8 transmission in Greece. We have also previously found that 48% of the HIV-1-positive and 56% of the HEPS subjects in Greece tested positive for anti-HHV-8 antibodies, a prevalence which is higher than that reported in other countries of Western Europe [7]. All the above mentioned data triggered the study of the seroprevalence of HHV-8 in healthy blood donors in Greece. It has been previously reported that the prevalence of HHV-8 in the general population in Greece is 7.6% and that the seropositivity for HHV-8 was associated with a history of endoscopic procedures and HBsAg positivity [8]. Nevertheless, we found no blood donor tested positive for HHV-8 IgG antibodies. We have used an ELISA with similar sensitivity detecting HHV-8 IgG antibodies [9] with the previous study and thus the discrepancy between the two studies could be attributed either to the relatively small size of our study or the differences between the general population and the blood donors population. We cannot overlook the fact that with the help of the donor screening questionnaire we practically exclude candidate donors with risk factors for HHV-8 transmission. Endoscopic procedures in the 6 months preceding blood donation, HBsAg seropositivity, and high-risk sexual behaviour are exclusion criteria for a blood donor.

In terms of HHV-6, the seroprevalence of HHV-6 in blood donors in Greece is unknown. Existing literature deals only with the detection of HHV-6 in the sera or tissue from patients with certain disease entities [10–14]. We detected HHV-6 IgG antibodies in 78.75% of blood donors, a seroprevalence similar to the one reported from other countries. Furthermore, the distribution between sexes and among age groups confirms the fact that infection with HHV-6 occurs early in life and that the IgG antibodies persist for a long time.

Blood transfusion–related transmission of herpesviruses from chronically infected donors to previously uninfected or immunocompromised recipients has been a subject of investigation for several years. The possibility of transfusion-related transmission of herpesviruses from healthy adult blood donors is moderately high for HHV-6, while it seems to be very low for HHV-8 [15].

A number of studies have shown that even in blood donors, who tested positive for HHV-8, HHV-8 DNA was rarely detected. Moreover, it has been suggested that leukoreduction could efficiently remove HHV-8 from blood donations, although some authors argue that this is true only in terms of the viral cellular counterpart. Cell-free HHV-8 was still present in a ranging percentage in 42% of subjects after filtration, as 1% to 20% of the total virus was not removed [16]. Furthermore, it seems that transfusion with fresh blood from HHV-8 positive blood donors can be associated with an increased risk of death [17]. But the fact, that no blood donor was positive for anti-HHV-8 IgG antibodies indicates that the risk for HHV-8 transmission with transfusion in Greece, if any, is negligible and does not warrant broad testing for HHV-8.

Regarding HHV-6 transmission with transfusion, the natural course of transfused WBCs carrying latent integrated HHV-6 in a immunocompetent recipient is the elimination of infected donor cells. However, in immunodeficient patients, especially those who receive stem cell transplants, there is an existing possibility that the integrated virus in the transplanted hematopoietic cells can be reactivated and lead to acute infection [18].

Although HHV-6 seropositivity reported from several studies is high, HHV-6 DNA is detected only in a small proportion of seropositive subjects. The possibility of HHV-6 transmission from a healthy blood donor with high HHV-6 viral load to an immunocompromised recipient cannot be excluded but it does not warrant broad testing for HHV-6. Further studies are needed, to clarify the potential risk of HHV-6 transmission from seropositive donors especially with high HHV-6 viral load but after the adoption of a common accredited detection method [17].

## Conclusions

In our study no blood donor tested positive for HHV-8 antibodies and the HHV-8 seroprevalence in the general population in Greece is not confirmed in our blood donor population. This may reflect, the usefulness of the donor screening questionnaire that tends to exclude donors with risk factors and/or behaviour which are related to HHV-8 transmission. Accordingly the risk for HHV-8 transmission related to transfusion in Greece, if any, is substantially low and does not warrant broad testing for HHV-8. Further studies are needed, in order to clarify the potential risk of HHV-6 transmission.
